# Diurnal Patterns of Soluble Amyloid Precursor Protein Metabolites in the Human Central Nervous System

**DOI:** 10.1371/journal.pone.0089998

**Published:** 2014-03-19

**Authors:** Justyna A. Dobrowolska, Tom Kasten, Yafei Huang, Tammie L. S. Benzinger, Wendy Sigurdson, Vitaliy Ovod, John C. Morris, Randall J. Bateman

**Affiliations:** 1 Department of Neurology, Washington University School of Medicine, St. Louis, Missouri, United States of America; 2 Department of Pathology and Immunology, Washington University School of Medicine, St. Louis, Missouri, United States of America; 3 Department of Radiology, Washington University School of Medicine, St. Louis, Missouri, United States of America; 4 Charles F. and Joanne Knight Alzheimer's Disease Research Center, Washington University School of Medicine, St. Louis, Missouri, United States of America; 5 Hope Center for Neurological Disorders, Washington University School of Medicine, St. Louis, Missouri, United States of America; Graduate School of Pharmaceutical Sciences, The University of Tokyo, Japan

## Abstract

The amyloid-β (Aβ) protein is diurnally regulated in both the cerebrospinal fluid and blood in healthy adults; circadian amplitudes decrease with aging and the presence of cerebral Aβ deposits. The cause of the Aβ diurnal pattern is poorly understood. One hypothesis is that the Amyloid Precursor Protein (APP) is diurnally regulated, leading to APP product diurnal patterns. APP in the central nervous system is processed either via the β-pathway (amyloidogenic), generating soluble APP-β (sAPPβ) and Aβ, or the α-pathway (non-amyloidogenic), releasing soluble APP-α (sAPPα). To elucidate the potential contributions of APP to the Aβ diurnal pattern and the balance of the α- and β- pathways in APP processing, we measured APP proteolytic products over 36 hours in human cerebrospinal fluid from cognitively normal and Alzheimer's disease participants. We found diurnal patterns in sAPPα, sAPPβ, Aβ_40_, and Aβ_42_, which diminish with increased age, that support the hypothesis that APP is diurnally regulated in the human central nervous system and thus results in Aβ diurnal patterns. We also found that the four APP metabolites were positively correlated in all participants without cerebral Aβ deposits. This positive correlation suggests that the α- and β- APP pathways are non-competitive under normal physiologic conditions where APP availability may be the limiting factor that determines sAPPα and sAPPβ production. However, in participants with cerebral Aβ deposits, there was no correlation of Aβ to sAPP metabolites, suggesting that normal physiologic regulation of cerebrospinal fluid Aβ is impaired in the presence of amyloidosis. Lastly, we found that the ratio of sAPPβ to sAPPα was significantly higher in participants with cerebral Aβ deposits versus those without deposits. Therefore, the sAPPβ to sAPPα ratio may be a useful biomarker for cerebral amyloidosis.

## Introduction

Alzheimer's disease (AD) is the most common neurodegenerative disorder, affecting an estimated 30 million people worldwide [1]. Although the pathophysiology of this disease is incompletely understood, the study of brain and cerebrospinal fluid (CSF) proteins, such as amyloid-β (Aβ) and tau, have provided insight into AD molecular pathophysiology [2]–[6]. The study of Aβ production, transport, and clearance is important for insight into normal brain protein handling and also for the pathophysiology of AD.

The first studies of Aβ concentrations over time indicated that CSF concentrations were sinusoidal over 24 hours in younger healthy participants [7] and suggested a possible circadian pattern. Subsequent studies in humans and animal models [8] demonstrated Aβ concentrations in the brain could be regulated by sleep/wake cycles and orexin. We reported that Aβ exhibits a diurnal pattern in both CSF [9] and blood [10] in healthy adults. The diurnal patterns, as determined by circadian amplitude, decreased with aging and amyloidosis. The immediate mechanism for diurnal regulation of Aβ has not been previously described, and possible causes for the Aβ diurnal pattern include, but are not limited to, diurnal regulation of Amyloid Precursor Protein (APP) transcription, translation, or transport, or diurnal regulation affecting the two secretases (β-secretase or γ-secretase) that cleave APP to produce Aβ. In this study, we evaluated the temporal relationship of Aβ with other proteolytic products of APP to inform about the cause of Aβ diurnal patterns in the CNS of healthy young and elderly humans, as well as those with amyloid pathology.

Amyloid precursor protein is a single-pass transmembrane protein processed through at least two pathways in the CNS: the β- (amyloidogenic) pathway and the α- (non-amyloidogenic) pathway [11]. This protein is cleaved in the amyloidogenic pathway by β-secretase releasing a soluble extracellular fragment called soluble APPβ (sAPPβ) [12]–[14]. The APP endodomain, C-terminal fragment 99 (CTF99), which remains in the transmembrane, is subsequently cleaved by γ-secretase, resulting in the generation of Aβ and the APP Intra-Cellular Domain (AICD). The non-amyloidogenic processing of APP occurs when α-secretase cleaves APP, producing soluble APPα (sAPPα). The endodomain of APP (CTF83) may then be cleaved by γ-secretase, resulting in the release of a fragment, p3. The formation of Aβ is precluded by α-secretase cleavage.

To further elucidate the potential contributions of APP to the Aβ diurnal pattern and the balance of the α- and β- pathways in APP processing, we measured APP proteolytic products sAPPβ, sAPPα, Aβ_40_, and Aβ_42_ over 36 hours in CSF from cognitively normal young and elderly participants, as well as in CSF from participants with AD.

## Materials and Methods

### Ethics statement

All human studies were approved by the Washington University Human Studies Committee and the General Clinical Research Center (GCRC) Advisory Committee. Written, informed consent was obtained from all participants prior to their enrollment in this study.

### Study design

Participants were recruited from the general public or through Washington University's Charles F. and Joanne Knight Alzheimer's Disease Research Center (Knight ADRC). All participants were in good general health. These participants were divided into three groups by age and brain amyloid status: 1) an Amyloid+ group of participants greater than 60 years of age and with probable amyloid plaques in the brain. Amyloid plaque status was determined by positron emission tomography using Pittsburgh compound B (PET PiB) or determined by an Aβ_42_ CSF mean concentration less than 350 pg/mL; 2) an Amyloid− age-matched group with no probable amyloid plaques in the brain as measured by PET PiB or determined by an Aβ_42_ CSF mean concentration greater than 350 pg/mL; 3) a Young Normal Control (YNC) group (18–50 years of age) that are likely PET PiB- [15]. PiB binds to fibrillar amyloid plaques in the brain [16]. A mean cortical binding potential (MCBP) was calculated for each participant to determine PET PiB (Amyloid) “+” or “−” status [15]. To measure the MCBP, binding potentials of PiB were averaged from specific brain regions: prefrontal cortex, precuneus, lateral temporal cortex, and gyrus rectus. MCBP scores of 0.18 or greater were designated as amyloid plaque positive (Amyloid**+**), while those less than 0.18 were designated as amyloid plaque negative (Amyloid**−**) [15]. Some participants did not have reported MCBP values, and, in those cases, a surrogate marker of amyloid deposition was used to assign the participant group. This surrogate marker was a low CSF Aβ_42_ concentration which has been shown to be inversely correlated with PET PiB measurements [17]. A CSF Aβ_42_ concentration was considered low (and the participant classified as Amyloid+) if it was detected as less than 350 pg/mL from an Aβ_42_ ELISA that used 21F12 (anti-Aβ_42_) as the capture antibody and biotinylated 3D6 antibody (directed against Aβ_1–5_) as the detection antibody.

### Demographics of study participants

A total of 49 participants (both men and women) were assessed in at least one part of this study. Specific sample size in each group varied depending on the experiment, and sample size for each group when diurnal patterns were observed is listed in the cosinor analyses section of the Methods. For the part of this study where APP metabolites were measured in a single CSF time point, there were 15 participants in the YNC group, 15 in Amyloid−, and 18 in Amyloid+.

The mean (SD) age for each participant group when all 49 participants were taken into account: YNC = 37.11 (±8.71) years; Amyloid− = 69.6 (±4.5) years; and Amyloid+: 76.3 (±7.5) years. Clinical Dementia Rating (CDR) at study onset was available for all participants. Of the Amyloid− participants, 33.3% had a CDR score greater than zero (exhibited cognitive deficits). Of the Amyloid+ participants, 29.4% had a CDR score equal to zero. All YNC subjects were free from any cognitive deficits.

### Sample collection and storage

Sample collection and handling were done as previously described [18]. Briefly, for all participants an intrathecal lumbar catheter was placed between the L3 and L4 interspace or the L4 and L5 interspace between 7:30 A.M. and 9:00 A.M. Collection of CSF began between 8:00 A.M. and 9:30 A.M. Every hour for 36 hours, 6 mL of CSF and 12 mL of plasma were withdrawn. Aliquots of CSF (1 mL) were immediately frozen at −80°C in Axygen maximum-recovery polypropylene tubes.

### Sample and standard handling

Aliquots (1 mL) from even hours with two freeze-thaw cycles were measured by sAPPα and sAPPβ ELISA. The effect of two freeze-thaw cycles was determined to not significantly change sAPPα and sAPPβ concentrations. Before plating, CSF samples were diluted in phosphate buffered saline-0.05% Tween20 (PBS-T) 75- to 150-fold for sAPPα, and 10- to 25-fold for sAPPβ. Recombinant standards from *E.coli* were used for both sAPPα (Sigma-Aldrich; St. Louis, MO) and sAPPβ (Sigma-Aldrich; St. Louis, MO). The concentration of the standards ranged from 1.6–75 ng/mL for sAPPα and 2.7–125 ng/mL for sAPPβ. Single freeze-thaw CSF aliquots from both odd and even hours were thawed on ice for the Aβ_40_ and Aβ_42_ ELISAs. They were diluted in a final buffer consisting of 2 mg/mL BSA (bovine serum albumin (Sigma-Aldrich; St. Louis, MO))-PBS-T, 3 M Tris, 10% Azide, 1× protease inhibitor cocktail. Each CSF and standard sample was assessed in triplicate.

### sAPPα ELISA protocol

For the sAPPα ELISAs, 96-well Nunc MaxiSorp flat bottom ELISA plates (eBiosciences, Inc.; San Diego, CA) were coated with 100 µL per well of 5 µg/mL of 8E5 (a monoclonal antibody raised to a bacterially expressed fusion protein corresponding to human APP_444–592_ of the APP_770_ transcript [19], courtesy of Eli Lilly). Plates were incubated for 24 hours on a shaker at 4°C, and then blocked with 3% dry milk in PBS-T for 1 hour 20 minutes at 37°C. To avoid plate position effects, samples were randomly assigned to a well on the plate. Secondary (detection) antibody (50 µL of 1∶10,000 6E10 [20], a monoclonal antibody reactive to Aβ_1–16_, otherwise known as APP_672–687_ (in the APP_770_ transcript), and having the epitope at Aβ_3–8_, or APP_674–679_) (Signet Covance; Dedham, MA) was added to each well. Samples and secondary antibody were incubated on a shaker at 4°C for 24 hours. Plates were washed 5 times with PBS-T and then Streptavidin Poly-HRP20 (Fitzgerald Industries International; Acton, MA), diluted at 1∶15,000 in 1% BSA-PBS-T, was added to each well at 100 µL/well. Plates were incubated in the dark for 1 hour at 37°C on a shaker. Plates were then washed 5 times with PBS-T and 5 times with PBS. The plates were developed as described for the sAPPβ ELISA below.

To test the specificity of the sAPPα assay, we ran a titration curve of sAPPα and sAPPβ protein standards on the same ELISA. The results demonstrated that this assay was specific for sAPPα and there was no detectable cross-reactivity with sAPPβ, as even the highest sAPPβ standard (300 ng/mL) did not produce an OD value above zero (Figure S1). The diluted CSF OD values fell within a linear range of the sAPPα standard curve

### sAPPβ ELISA protocol

For the sAPPβ ELISA, 96-well Nunc MaxiSorp flat bottom ELISA plates (eBiosciences, Inc.; San Diego, CA) were coated with 100 µL per well of 10 µg/mL of the monoclonal antibody, 8E5. Plates were incubated for 24 hours on a shaker at 4°C and subsequently blocked with 3% dry milk in PBS-T for 1 hour 20 minutes at 37°C. Samples were randomly assigned a plate well position and incubated for 24 hours on a shaker at 4°C. They were then washed 5 times with PBS-T. An antibody against the neo-epitope of sAPPβ (APP_670/671_ of the APP_770_ transcript) (courtesy of Eli Lilly) was used as the secondary (detection) antibody at a volume of 50 µL and a concentration of 0.5 µg/µL, diluted in PBS-T pre-warmed to 37°C. The sAPPβ detection antibody was added to each well and incubated at 37°C for 90 minutes. Plates were washed 10 times with PBS-T, and 100 µL Streptavidin Poly-HRP40 (Fitzgerald Industries International; Acton, MA), diluted at 1∶20,000 in 1% BSA-PBS-T, was added to each well. Plates were incubated in the dark for 1 hour at 25°C on a shaker and washed 5 times with PBS-T and 5 times with PBS. For the sAPPα and sAPPβ ELISAs, 100 µL/well of ELISA TMB Super Slow (Sigma-Aldrich; St. Louis, MO), pre-warmed to 25°C, was then added to each well. Optical density (OD) was measured at 650 nm using a Biotek Synergy 2 plate reader after 5–30 minutes.

We tested the specificity of the sAPPβ assay by running a titration curve of the sAPPβ and sAPPα protein standards on the same ELISA. The results demonstrated that this assay was specific for sAPPβ and that cross-reactivity with sAPPα was negligible. The OD value for the sAPPβ standard of 8.5 ng/mL was approximately the same as that for the sAPPα standard of 300 ng/mL (Figure S2). This indicated that this ELISA was approximately 35-fold more selective for sAPPβ than for sAPPα. The diluted CSF OD values fell within a linear range of the sAPPβ standard curve and well above the highest sAPPα standard's (300 ng/mL) OD value. Given that in biological samples sAPPα and sAPPβ were nearly equal in molar concentrations, this minimal cross-reactivity of sAPPα in the sAPPβ ELISA was negligible. Thus, we concluded that any fluctuations we observed in sAPPβ levels using this ELISA were attributed solely to sAPPβ, and not to sAPPα.

### Aβ_40_ and Aβ_42_ ELISA protocols

Corning 96-well half area clear flat bottom polystyrene high bind ELISA plates (Corning Life Sciences, Tewksbury, MA) were coated with 1.25 µg/mL HJ7.4 (Aβ_37–42_) or 2.5 µg/mL HJ2 (Aβ_33–40_) in PBS plus 20% glycerol (PBS-G), then incubated 1 hour at 25°C followed by overnight incubation at 4°C. The next day the plates were blocked with 2% BSA-PBS-T for 90 minutes at 4°C. Samples were randomly assigned a well on the plate. Diluted CSF samples and standards were pipetted at a volume of 50 µL per well onto freshly washed plates. The samples were loaded in triplicate and incubated overnight at 4°C. After incubation and washing, the plates were incubated for 90 minutes at 25°C with 0.2 µg/mL HJ5.1-Biotin (Aβ_13–28_) in 1% BSA-PBS-T-G. Plates were then washed three times with 190 µL PBS-T, followed by incubation in Streptavidin Poly-HRP40 (Fitzgerald Industries International; Acton, MA), diluted at 1∶12,000 in 1% BSA-PBS-T-G, for 90 minutes at 25°C. Plates were subsequently washed three times with 190 µL PBS-T. They were then incubated with 50 µL/well of Slow ELISA TMB (pre-warmed to 25°C) for 5–30 minutes. Optical density (OD) was read at 650 nm using a Biotek Synergy 2 plate reader.

### CSF protein level quantification

Soluble APPα, sAPPβ, Aβ_40_, and Aβ_42_ concentration levels were quantified using the Biotek Gen5 software (version #1.08.4) based on the non-linear five parametric standard curves generated from the recombinant sAPPα, sAPPβ, Aβ_40_, and Aβ_42_ standards. The OD values of the CSF samples fell within the linear range of the standard curve and were converted to concentration levels. The product of the concentration and the dilution factor was calculated in order to determine the final CSF concentration of each protein.

Total protein levels of each sample were measured by BCA assay (Thermo Fisher Scientific, Inc.; Rockford, IL) as previously reported [9]. The intra-sample coefficient of variation mean was 2% for duplicates.

### Group-averaged cosinor analyses

Serial sAPPα and sAPPβ concentrations were binned in two hour increments as samples were from every other hour. Serial Aβ_40_ and Aβ_42_ concentrations were left unbinned because hourly concentrations were measured. For each APP metabolite, each participant's hourly metabolite's concentration was normalized to that metabolite's mean concentration over 36 hours. The normalized value was calculated as a percentage of each participant's mean (100×value/mean). Hourly (Aβ_40_ and Aβ_42_) and bi-hourly (sAPPα and sAPPβ) concentrations of each metabolite were averaged among all participants in each participant group to produce normalized mean 36 hour concentrations. Next, the linear concentration rise over time that was observed in each metabolite was subtracted out of the mean concentrations and a single cosinor fit was applied for each metabolite as described previously [9]. Briefly, a cosine transformation was applied to the time variable using 24 hours as the default circadian cycle, and Graphpad Prism version 5.01 for Windows (GraphPad Software; San Diego, CA) was used to estimate the parameters of the circadian rhythms for each metabolite. The amplitude (distance between the peak to the midline of the cosine wave) was determined for each participant group. For all cosinor analyses, the YNC group consisted of 13 participants. The Amyloid**−** group included 19 participants for sAPPα and sAPPβ cosinor analyses, and 15 participants for Aβ_40_ and Aβ_42_ cosinor analyses. The Amyloid+ group had 17 participants for sAPPα and sAPPβ cosinor analyses, and 14 participants for Aβ_40_ and Aβ_42_ cosinor analyses.

### Individual cosinor analyses

For each participant, sAPPα, sAPPβ, Aβ_40_, and Aβ_42_ levels over 36 hours were analyzed using a single cosinor analysis as described above. Mesor (midline of the metabolite oscillation), amplitude (distance between the peak and mesor), amplitude-to-mesor ratio, and acrophase (time at which the peak occurs) were calculated for each metabolite for each participant. Then, participant group means for each of the metabolites' cosinor parameters were determined. Group sample size for these analyses was the same as for the group-averaged cosinor analyses.

### Statistical analyses

Analyses were performed using Microsoft Office Excel 2007 and GraphPad Prism version 5 for Windows (GraphPad Software, San Diego, California, USA). Student's *t*-tests and ANOVAs were used to determine whether there were differences in cosinor parameters between groups. 95% confidence intervals were reported. Correlations between APP metabolites were measured by calculating the correlation coefficient (Pearson r values reported). Soluble APPα, sAPPβ, and sAPPβ/α ratio were compared among groups using a student's *t*-test and ANOVA. 95% confidence intervals were reported.

## Results

### Circadian patterns of APP metabolites

In order to determine APP processing over time within the same participant, temporal CSF samples from a particular participant were randomly assigned a well position on four sandwich ELISAs: specific for sAPPα, sAPPβ, Aβ_40_, or Aβ_42_. This allowed for analysis of APP metabolite concentrations in the CSF over time. To compare age and amyloid deposition effects on hourly dynamics of APP metabolites, the Young Normal Control (YNC) group was compared to the Amyloid− and Amyloid+ groups.

### sAPPα and sAPPβ exhibit circadian patterns

**Figure 1:**
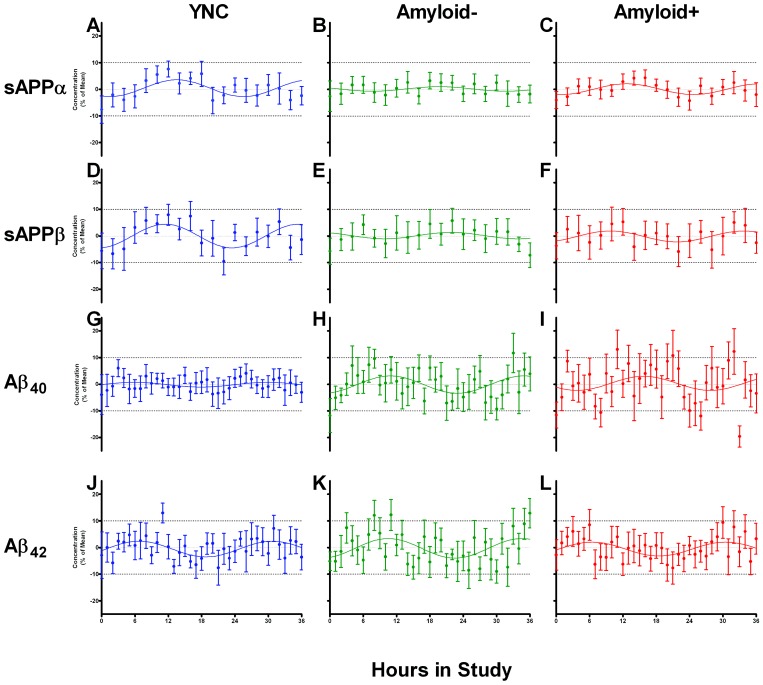
Group-averaged diurnal rhythms of four APP metabolites are present. Cosinor fits were applied to each participant group's percentage of the mean for 36 hours of a particular APP metabolite's concentration. This was done after adjusting for each participant's individual baseline and subtracting out the group's linear increase in concentration over time. Results from all three participant groups are reported for sAPPα (**A–C**), sAPPβ (**D–F**), Aβ_40_ (**G–I**), and Aβ_42_ (**J–L**).

Cerebrospinal fluid sAPPα and sAPPβ hourly concentrations had significant fits to a 24 hour cosinor pattern in the YNC group. The average amplitude of the diurnal pattern for sAPPα was 2.9%±1.3% (SEM) (Figure 1A). For sAPPβ, the average amplitude was 4.4%±1.6% (SEM) (Figure 1D).

### Group-averaged sAPPα and sAPPβ circadian amplitudes lower with older age

When a 24 hour cosine curve was fit to the three group-averaged sAPPα hourly concentrations, the YNC group exhibited an amplitude that significantly deviated from zero (2.9%) and was significantly greater than the Amyloid**−** (0.9%) and Amyloid**+** (2%) groups, which both did not deviate significantly from zero (Figure 1A–C). A similar trend was observed when a cosine curve was fit to the three group-averaged sAPPβ hourly concentrations (Figure 1D–F). Amplitude of sAPPβ for the YNC group was 4.4%, Amyloid**−** was 1.2%, and Amyloid**+** was 2%. Only the sAPPβ amplitude of the YNC group significantly deviated from zero. Amplitude of Aβ_40_ for the YNC group was 0.9%, Amyloid**−** was 3.2%, and Amyloid**+** was 2.6% (Figure 1G–I). Only the Aβ_40_ amplitude of the Amyloid− group significantly deviated from zero. Amplitude of Aβ_42_ for the YNC group was 2.9%, Amyloid**−** was 3.8%, and Amyloid**+** was 0.4% (Figure 1J–L). Only the Aβ_42_ amplitude of the YNC group significantly deviated from zero.

### Individual sAPPα and sAPPβ amplitude-to-mesor values decrease with age; Aβ_40_ and Aβ_42_ amplitude-to-mesor values unchanged

**Figure 2:**
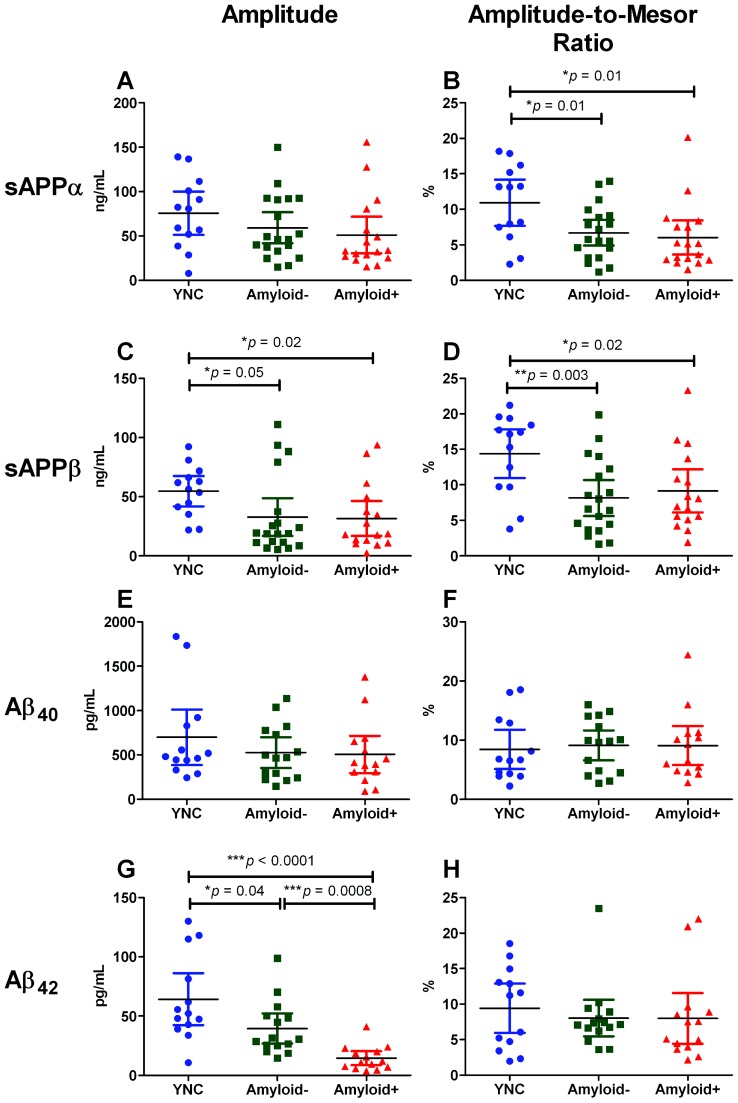
Circadian rhythm parameters of four APP metabolites in YNC, Amyloid−, and Amyloid+ groups. **A**) Group-averaged sAPPα amplitudes were not significantly different among groups. **B**) The sAPPα amplitude-to-mesor ratio was highest in YNC and significantly lower in Amyloid**−** (*p = 0.01) and Amyloid**+** (*p = 0.01). There was no significant difference between the Amyloid**−** and Amyloid**+** groups (*p* = 0.6). **C**) Group-averaged sAPPβ amplitudes were significantly higher in YNC than in Amyloid− (*p = 0.05) and Amyloid+ (*p = 0.02). **D**) The sAPPβ amplitude-to-mesor ratio was highest in YNC and significantly lower in Amyloid**−** (**p = 0.003) and Amyloid**+** (*p = 0.02). There was no significant difference between the Amyloid**−** and Amyloid**+** groups (*p* = 0.6). **E**) Group-averaged Aβ_40_ amplitude values were not significantly different among any of the participant groups. **F**) Amplitude-to-Mesor ratio for Aβ_40_ was also not significantly different among groups. **G**) Group-averaged Aβ_42_ amplitudes were significantly highest in YNC when compared to Amyloid− (*p = 0.04) and Amyloid+ (***p<0.0001). The Amyloid− group also had a significantly higher Aβ_42_ amplitude than the Amyloid+ group (***p = 0.0008). **H**) The Aβ_42_ amplitude-to-mesor ratios did not differ significantly among groups.

To control for differences in average values of amplitude and mesor among participants, the amplitude-to-mesor ratios were calculated for each group. In the YNC group, sAPPα amplitude-to-mesor ratio was, on average, 10.93% (min.: 2.3%, max.: 18.2%). Both the Amyloid**−** (6.7%; Min: 1.2%, max.: 14.0%; *p = 0.01) and Amyloid**+** (6.0%; min.: 1.5%, max.: 20.1%; *p = 0.01) groups had significantly lower sAPPα amplitude-to-mesor ratios than YNC. There was no significant difference between the Amyloid**−** and Amyloid**+** groups (*p* = 0.6) (Table 1; Figure 2B).

**Table 1:** Comparison of Cosinor Parameters for sAPPα among 3 groups.

Participant Group	Amplitude, ng/mL Mean (SD)	Mesor, ng/mL Mean (SD)	Amplitude-to-Mesor Ratio, % Mean (SD)	Acrophase (h) Mean (SD)
YNC (n = 13)	75.74 (11.15)	731.0 (86.65)	10.93 (1.5)	3.9 (5.6)
Amyloid− (n = 19)	59.24 (8.317)	1100 (159.4)	6.7 (0.87)	2.7 (5.3)
Amyloid+ (n = 17)	51.1 (9.734)	898.1 (72.95)	6.04 (1.14)	3.9 (6.2)

Abbreviations: **YNC**: participants classified as young (cognitively) normal healthy controls; **Amyloid−**: participants with a Mean Cortical Binding Potential (MCBP) less than 0.18, or, in the absence of MCBP measurements, a mean CSF Aβ_42_ concentration greater than 350 pg/mL; **Amyloid+**: participants with MCBP greater than or equal to 0.18, or, in the absence of MCBP measurements, a mean CSF Aβ_42_ concentration less than 350 pg/mL.

Similar trends were observed among groups when sAPPβ amplitude-to-mesor ratio was measured. In YNC, the mean sAPPβ amplitude-to-mesor ratio was 14.38% (min.: 3.8%, max.: 21.2%). The Amyloid**−** (8.15%; min.: 1.7%, max.: 19.9%; **p = 0.003) and Amyloid**+** (9.16%; min.: 1.9%, max.: 23.3%; *p = 0.02) groups had significantly lower sAPPβ amplitude-to-mesor ratios than YNC. However, Amyloid**−** and Amyloid**+** groups did not significantly differ from one another (*p* = 0.6) (Table 2; Figure 2D).

**Table 2:** Comparison of Cosinor Parameters for sAPPβ among 3 groups.

Participant Group	Amplitude, ng/mL Mean (SD)	Mesor, ng/mL Mean (SD)	Amplitude-to-Mesor Ratio, % Mean (SD)	Acrophase (h) Mean (SD)
YNC (n = 13)	54.61 (5.9)	416.5 (50.39)	14.38 (1.58)	1.5 (2.0)
Amyloid− (n = 19)	32.78 (7.66)	383.2 (47.76)	8.15 (1.21)	1.5 (2.4)
Amyloid+ (n = 17)	31.57 (6.95)	344.3 (55.27)	9.16 (1.42)	3.5 (6.2)

Abbreviations: **YNC**: participants classified as young (cognitively) normal healthy controls; **Amyloid−**: participants with a Mean Cortical Binding Potential (MCBP) less than 0.18, or, in the absence of MCBP measurements, a mean CSF Aβ_42_ concentration greater than 350 pg/mL; **Amyloid+**: participants with MCBP greater than or equal to 0.18, or, in the absence of MCBP measurements, a mean CSF Aβ_42_ concentration less than 350 pg/mL.

On the contrary, the Aβ_40_ amplitude-to-mesor ratio was not statistically different among all three groups. In YNC, the mean Aβ_40_ amplitude-to-mesor ratio was 8.46% (min.: 2.2%, max.: 18.5%). The Amyloid**−** group had a mean Aβ_40_ amplitude-to-mesor ratio of 9.13% (min.: 2.7%, max.: 16%) and the Amyloid**+** group had a mean Aβ_40_ amplitude-to-mesor ratio of 9.09% (min.: 2.8%, max.: 24.4%). None of these groups' Aβ_40_ amplitude-to-mesor ratios were significantly different from one another (YNC vs. Amyloid**−**: *p* = 0.7; YNC vs. Amyloid**+**: *p* = 0.8; Amyloid**−** vs. Amyloid**+**: *p* = 0.99) (Table 3; Figure 2F).

**Table 3:** Comparison of Cosinor Parameters for Aβ_40_ among 3 groups.

Participant Group	Amplitude, pg/mL Mean (SD)	Mesor, pg/mL Mean (SD)	Amplitude-to-Mesor Ratio, % Mean (SD)	Acrophase (h) Mean (SD)
YNC (n = 13)	698.8 (143.8)	8966 (936.1)	8.46 (1.52)	6.7 (7.1)
Amyloid− (n = 15)	526.3 (80.29)	6373 (762)	9.13 (1.18)	7.1 (6.9)
Amyloid+ (n = 14)	505.5 (97.67)	5872 (795.8)	9.09 (1.5)	8.2 (8.1)

Abbreviations: **YNC**: participants classified as young (cognitively) normal healthy controls; **Amyloid−**: participants with a Mean Cortical Binding Potential (MCBP) less than 0.18, or, in the absence of MCBP measurements, a mean CSF Aβ_42_ concentration greater than 350 pg/mL; **Amyloid+**: participants with MCBP greater than or equal to 0.18, or, in the absence of MCBP measurements, a mean CSF Aβ_42_ concentration less than 350 pg/mL.

When Aβ_42_ amplitude-to-mesor ratio was measured, similar trends to the Aβ_40_ amplitude-to-mesor ratios were observed. In YNC, the mean Aβ_42_ amplitude-to-mesor ratio was 9.43% (min.: 1.9%, max.: 18.5%). The Amyloid**−** group had a mean Aβ_42_ amplitude-to-mesor ratio of 8.04% (min.: 3.6%, max.: 23.5%) and the Amyloid**+** group had a mean Aβ_42_ amplitude-to-mesor ratio of 7.99% (min.: 2.2%, max.: 22%). None of these groups' Aβ_42_ amplitude-to-mesor ratios were significantly different from one another (YNC vs. Amyloid**−**: *p* = 0.5; YNC vs. Amyloid**+**: *p* = 0.5; Amyloid**−** vs. Amyloid**+**: *p* = 0.98) (Table 4; Figure 2H).

**Table 4:** Comparison of Cosinor Parameters for Aβ_42_ among 3 groups.

Participant Group	Amplitude, pg/mL Mean (SD)	Mesor, pg/mL Mean (SD)	Amplitude-to-Mesor Ratio, % Mean (SD)	Acrophase (h) Mean (SD)
YNC (n = 13)	64.26 (10.06)	830.7 (117.6)	9.43 (1.59)	2.9 (2.7)
Amyloid− (n = 15)	39.49 (5.9)	518.6 (54.08)	8.04 (1.2)	1.7 (1.8)
Amyloid+ (n = 14)	14.5 (2.73)	206.9 (26.74)	7.99 (1.65)	5.0 (6.2)

Abbreviations: **YNC**: participants classified as young (cognitively) normal healthy controls; **Amyloid−**: participants with a Mean Cortical Binding Potential (MCBP) less than 0.18, or, in the absence of MCBP measurements, a mean CSF Aβ_42_ concentration greater than 350 pg/mL; **Amyloid+**: participants with MCBP greater than or equal to 0.18, or, in the absence of MCBP measurements, a mean CSF Aβ_42_ concentration less than 350 pg/mL.

### Individual Aβ_42_ amplitude values decrease with age and amyloidosis, as sAPPβ amplitude decreases with age; sAPPα and Aβ_40_ amplitudes are not significantly different among groups

On average, for YNC the sAPPα amplitude was 75.74 ng/mL (min.: 7.7 ng/mL, max.: 139.1 ng/mL), in Amyloid**−** it was 59.24 ng/mL (min.: 15.1 ng/mL, max.: 149.7 ng/mL), and in Amyloid**+** it was 51.1 ng/mL (min.: 15.3 ng/mL, max.: 155.8 ng/mL). Although a trend toward a decrease of sAPPα amplitude with increase in age was observed, the groups were not significantly different by their sAPPα mean amplitudes (YNC vs. Amyloid**−**: *p* = 0.2; YNC vs. Amyloid**+**: *p* = 0.1; Amyloid**−** vs. Amyloid**+**: *p* = 0.5) (Table 1; Figure 2A).

However, with respect to sAPPβ mean amplitudes there was a significant difference between YNC and either the Amyloid**−** or the Amyloid**+** group. The sAPPβ mean amplitude in the YNC group was 54.61 ng/mL (min.: 21.8 ng/mL, max.: 92.2 ng/mL). The Amyloid**−** group had a mean sAPPβ amplitude that was 40% lower (32.78 ng/mL; min.: 5.4 ng/mL, max.: 111.1 ng/mL) than YNC (*p = 0.05), whereas the Amyloid**+** group had a mean sAPPβ amplitude that was 42% lower (31.57 ng/mL; min.: 2.4 ng/mL, max.: 93.7 ng/mL) than YNC (*p = 0.02). There was no significant difference in sAPPβ amplitude between the Amyloid**−** and Amyloid**+** groups (*p* = 0.9) (Table 2; Figure 2C).

For the YNC group, the mean Aβ_40_ amplitude was 698.8 pg/mL (min.: 287.3 pg/mL, max.: 1834 pg/mL). There was a trend for decreased mean Aβ_40_ amplitude with age. The Amyloid**−** group had a mean Aβ_40_ amplitude of 526.3 pg/mL (min.: 148.1 pg/mL, max.: 1138 pg/mL) and the Amyloid**+** group had a mean Aβ_40_ amplitude of 505.5 pg/mL (min.: 90.55 pg/mL, max.: 1381 pg/mL). This trend did not reach statistical significance (YNC vs. Amyloid**−**: *p* = 0.29; YNC vs. Amyloid**+**: *p* = 0.27; Amyloid**−** vs. Amyloid**+**: *p* = 0.89) (Table 3; Figure 2E).

In contrast, the mean Aβ_42_ amplitudes were significantly different among all groups. In the YNC the mean Aβ_42_ amplitude was 64.26 pg/mL (min.: 10.6 pg/mL, max.: 130.1 pg/mL). The Amyloid**−** group had a mean Aβ_42_ amplitude that was 39% lower (39.49 pg/mL; min.: 14.4 pg/mL, max.: 99 pg/mL) than the YNC group (*p = 0.04). The Amyloid**+** group had a mean Aβ_42_ amplitude that was 77% lower (14.5 pg/mL; min.: 3.7 pg/mL, max.: 41 pg/mL) than the YNC group (***p<0.0001) and 63% lower than the Amyloid**−** group (***p = 0.0008) (Table 4; Figure 2G).

### sAPPα and sAPPβ mesors unchanged while Aβ_40_ mesor decreases with age, and Aβ_42_ mesor decreases with age and amyloidosis

In YNC, sAPPα levels had a mean mesor over 36 hours of 731.0 ng/mL (min.: 250.4 ng/mL, max.: 1254 ng/mL). In Amyloid**−**, sAPPα levels displayed a mean mesor of 1100 ng/mL (min.: 191.5 ng/mL, max.: 2805 ng/mL). The Amyloid**+** group had a mean sAPPα mesor level of 898.1 ng/mL (min.: 386 ng/mL, max.: 1353 ng/mL). None of these groups' sAPPα mesors were significantly different from one another (YNC vs. Amyloid**−**: *p* = 0.2; YNC vs. Amyloid**+**: *p* = 0.08; Amyloid**−** vs. Amyloid**+**: *p* = 0.3) (Table 1).

The mean sAPPβ mesor in the YNC group was 416.5 ng/mL (min.: 229 ng/mL, max.: 928.3 ng/mL). This was not significantly different (*p* = 0.6) from the mean sAPPβ mesor in Amyloid**−** (383.2 ng/mL; min.: 100.5 ng/mL, max.: 831.9 ng/mL), nor from the mean sAPPβ mesor level in Amyloid**+** (344.3 ng/mL; min.: 117.5 ng/mL, max.: 899.8 ng/mL; *p* = 0.4). The mean sAPPβ mesors in the Amyloid**−** and Amyloid**+** groups were also not significantly different from one another (*p* = 0.6) (Table 2).

The YNC group had a mean Aβ_40_ mesor of 8966 pg/mL (min.: 2430 pg/mL, max.: 13433 pg/mL). The Amyloid**−** group had a 29% lower mean Aβ_40_ mesor (6373 pg/mL; min.: 1332 pg/mL, max.: 11089 pg/mL) than the YNC group (*p = 0.04). The Amyloid**+** group exhibited a 35% lower Aβ_40_ mesor (5872 pg/mL; min.: 1505 pg/mL, max.: 10768 pg/mL) than the YNC group (*p = 0.02). There was no statistically significant difference in mean Aβ_40_ mesor values between the Amyloid**−** and Amyloid**+** groups (*p* = 0.7) (Table 3).

The mean Aβ_42_ mesors were significantly different among all groups. On average, the YNC group's Aβ_42_ mesor was 830.7 pg/mL (min.: 255.7 pg/mL, max.: 1683 pg/mL). The Amyloid**−** group had a 38% lower mean Aβ_42_ mesor (518.6 pg/mL; min.: 195 pg/mL, max.: 885.3 pg/mL) than the YNC group (*p = 0.02). The Amyloid**+** group had a 75% lower mean Aβ_42_ mesor (206.9 pg/mL; min.: 48.85 pg/mL, max.: 471.3 pg/mL) than the YNC group (***p<0.0001) and a 60% lower mean Aβ_42_ mesor than the Amyloid**−** group (***p<0.0001) (Table 4).

### Individual acrophases are not significantly different with age or amyloidosis

There is much inter-subject variability within groups for each metabolite's acrophase. Thus, any differences in time at peak/trough among participant groups are not significantly different. Data are provided in Tables 1–4. In the case of all four metabolites, differences among average acrophase of participant groups never reached statistical significance (p>0.05). Differences among metabolites' group-averaged acrophases were not evaluated because when no significant cosinor fit is found (as in Figure 1B, C, E, F, G, I, K, L), the acrophase is not a valid parameter to compare groups.

### No diurnal pattern exhibited in total protein levels of Amyloid− and Amyloid+ groups

As a negative control for diurnal rhythms, we assayed total CSF protein over 36 hours using a micro BCA assay. Total protein data was only available for a subset of participants in each group. We measured that, on average, total protein concentrations were significantly lower in YNC as compared with the older participants (YNC = 797.2 µg/mL (n = 6), Amyloid− = 895.1 µg/mL (n = 6), and Amyloid+ = 871.4 µg/mL (n = 5), ***p<0.0001). A cosinor fit was applied to the mean of each group's total protein level. A significant cosinor fit was found in the YNC group, with an amplitude 4.5% (95% CI: −6.1% to −2.9%). Cosinor fits for both older groups were insignificant because the amplitudes' 95% CIs crossed zero: Amyloid− (95% CI: −1.4% to +8.6%) and Amyloid+ (95% CI: −8.4% to +1.4%) (Figure S3). Acrophase was calculated only for the YNC (1.1±0.7 h), as the other groups did not exhibit a significant cosinor fit. Owing to high inter-subject variability within the YNC group and approximately only 46% of participants having BCA data for analysis, we cannot conclude that a significant cosinor fit in the YNC group would hold up with a full dataset.

### sAPP and Aβ positively correlated, except in amyloidosis

**Figure 3:**
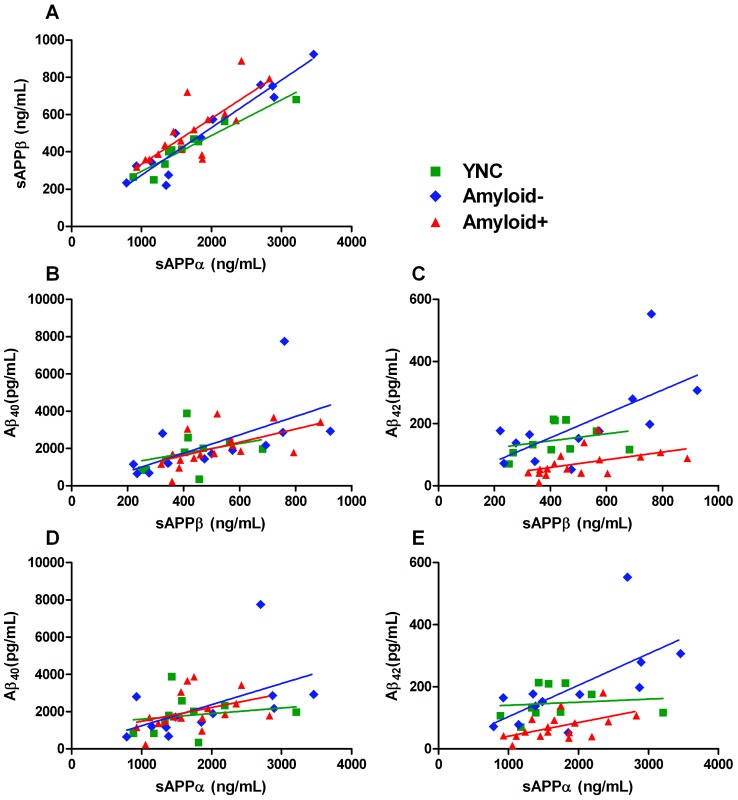
Correlations between APP metabolites. Amyloid Precursor Protein metabolites in the first CSF collection for each participant were correlated to determine relationships between the APP processing pathways. Each participant's cerebrospinal fluid sample was drawn between 7:30 A.M. and 9:00 A.M. The four APP metabolites' concentrations were measured using four separate metabolite-specific ELISAs, and, for each participant, plotted against one another. The correlation coefficient was then calculated for each group. **A**) sAPPα and sAPPβ concentrations for each participant were plotted against one another and showed a positive correlation in all groups (YNC: r = 0.95, ***p<0.0001; Amyloid−: r = 0.93, ***p<0.0001; Amyloid+: r = 0.86, **p = 0.0015). **B**) sAPPβ and Aβ_40_ concentrations for each participant were plotted against one another : YNC: r = 0.84, *p = 0.018; Amyloid−: r = 0.68, **p = 0.0048; Amyloid+: r = 0.25, *p* = 0.52). **C**) sAPPβ and Aβ_42_ concentrations for each participant were plotted against one another and a positive correlation was detected in YNC (r = 0.57, *p* = 0.18) and Amyloid− groups (r = 0.5, *p* = 0.14), but no correlation was detected in the Amyloid+ group (r = −0.08, *p* = 0.84). **D**) sAPPα and Aβ_40_ concentrations for each participant were plotted against one another and compared among the groups: YNC: r = 0.69, *p* = 0.09; Amyloid−: r = 0.84, **p = 0.0025; Amyloid+: r = 0.2, *p* = 0.6). **E**) sAPPα and Aβ_42_ concentrations for each participant were plotted against one another. The correlation results are as follows: YNC (r = 0.39, *p* = 0.38); Amyloid− (r = 0.64, *p* = 0.04); and Amyloid+ (r = −0.01, *p* = 0.97).

In order to determine the relationship of α- and β-secretases on APP processing, correlations of sAPPα, sAPPβ, Aβ_40_, and Aβ_42_ were calculated in CSF from a single time-point at the onset of the study (between 7:30 A.M. and 9:00 A.M.) in the three participant groups: YNC, Amyloid**−**, and Amyloid**+**. Soluble APPα and sAPPβ were positively correlated in all groups (YNC: r = 0.95, ***p<0.0001; Amyloid**−**: r = 0.93, ***p<0.0001; Amyloid**+**: r = 0.86, **p = 0.002) (Figure 3A). Soluble APPβ was positively correlated to Aβ_40_ in YNC (r = 0.84, *p = 0.02), and Amyloid**−** groups (r = 0.68, **p = 0.005), but not in the Amyloid**+** group (r = 0.25, *p* = 0.5) (Figure 3B). Soluble APPα was also positively correlated to Aβ_40_ in the Amyloid**−** group (r = 0.84, **p = 0.003), and trended toward a positive correlation in the YNC group (r = 0.69, *p* = 0.1). There was not any strong correlation between sAPPα and Aβ_40_ in the Amyloid**+** group (r = 0.2, *p* = 0.6) (Figure 3D). There was a trend for sAPPβ to be positively correlated to Aβ_42_ in YNC (r = 0.57, *p* = 0.2), and Amyloid**−** groups (r = 0.5, *p* = 0.1); but there was no correlation in the Amyloid**+** group (r = −0.08, *p* = 0.8) (Figure 3C). Similarly, sAPPα also trended to a positive correlation with Aβ_42_ in YNC (r = 0.39, *p* = 0.4) and Amyloid**−** groups (r = 0.64, *p* = 0.04); but not in the Amyloid**+** group (r = −0.01, *p* = 1.0) (Figure 3E).

### sAPPβ/sAPPα ratio is elevated in amyloidosis

**Figure 4:**
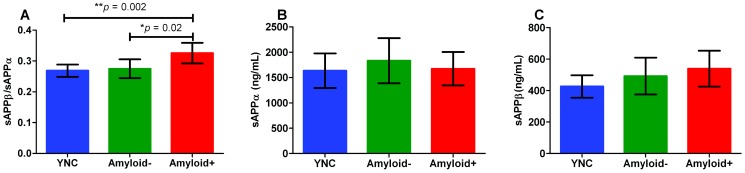
Separating participant groups by the sAPPβ/sAPPα ratio. We compared sAPPβ and sAPPα concentrations, as well as the sAPPβ/sAPPα ratio, among groups using the first CSF collection. Each participant's first CSF sample was drawn between 7:30 A.M. and 9:00A.M. sAPPβ and sAPPα concentrations were measured using two separate metabolite-specific ELISAs. Student's *t*-tests were used and graphs show 95% Confidence Interval error bars. **A**) sAPPβ/sAPPα ratio was higher with amyloid deposition (Amyloid**+**) as compared to healthy, older controls (Amyloid**−**) (*p = 0.02) or young healthy controls (YNC) (**p = 0.002). No significant difference was detected between the ratio of the YNC and Amyloid**−** groups (*p* = 0.6). **B**) sAPPα concentrations were not significantly higher in Amyloid**+** than in YNC (*p* = 1.0) or Amyloid**−** (*p* = 0.5). No significant difference was detected between the sAPPα concentration of the YNC and Amyloid**−** groups (*p* = 0.4). **C**) sAPPβ concentrations were not significantly higher in Amyloid**+** than in YNC (*p* = 0.09) nor Amyloid**−** (*p* = 0.6). No significant difference was detected between sAPPβ concentrations from the YNC and Amyloid**−** groups (*p* = 0.3).

In order to determine the effects of age and amyloidosis on the APP processing pathways, APP metabolites from a single CSF time-point at the onset of the study (between 8:00 A.M. and 10:00 A.M.) were compared among three participant groups: YNC, Amyloid**−**, and Amyloid**+**. The sAPPβ to sAPPα ratio was 0.26±0.01 (≈1∶3 ratio, n = 15) in YNC, and 0.26±0.02 (≈1∶3 ratio, n = 15) in Amyloid**−**. However, the ratio increased to 0.32±0.05 (≈1∶2 ratio, n = 10) for Amyloid**+**. The sAPPβ/sAPPα ratio was significantly higher in Amyloid**+** participants than in Amyloid**−** (*p = 0.02) and YNC (**p = 0.002) (Figure 4A). However, taken independently, mean sAPPα and sAPPβ concentrations were not significantly different among groups, suggesting that the sAPPβ/sAPPα ratio corrected for other variances which were not associated with amyloidosis (Figure 4B–C).

In order to determine if there was a similar pattern in sAPPβ/sAPPα ratio differences among groups when measurements were taken over a full 36 hour time-course (versus at a single time-point: hour 0), for each participant sAPPβ and sAPPα concentrations were individually averaged over 36 hours. Each participant's 36 hour averaged sAPPβ concentration and their respective 36 hour averaged sAPPα concentration were then used to determine the mean sAPPβ/sAPPα ratio. These mean ratios were then, in turn, averaged to determine a participant group average of the mean sAPPβ/sAPPα ratio. The mean sAPPβ to sAPPα ratio was 0.59±0.04 (n = 15) in YNC, which was significantly higher (*p = 0.03) than either the Amyloid− (n = 19) or the Amyloid+ (n = 17) ratio (both ratios were 0.42±0.06) (Figure S4A).

Additionally, each participant's sAPPβ mesor and sAPPα mesor were used to determine individual mesor sAPPβ/sAPPα ratios. The mesor sAPPβ to sAPPα ratio was 0.59±0.04 (n = 15) in YNC, which was significantly higher than the Amyloid− and Amyloid+ mesor ratios. Mesor ratio means and error for the two older groups were identical to averaged ratios and errors (Figure S4B).

The results from the mean sAPPβ to sAPPα ratio and the mesor sAPPβ to sAPPα ratio are almost identical because they represent nearly the same parameter. These results also contrast with the increased sAPPβ to sAPPα ratio with amyloidosis when only the first CSF sample collected (hour 0) is analyzed. The mean concentrations and the mesor are calculated from runs on multiple ELISA plates over many months and may not be directly comparable, while the hour 0 samples were run on the same plate and can be directly compared. Thus, we conclude the increased sAPPβ to sAPPα ratio in amyloidosis when measuring at hour 0 is most reliable as it avoids assay drift and also the modeling of the calculated mesor value.

## Discussion

We evaluated whether APP exhibited diurnal fluctuations similar to that of Aβ, which would help inform why Aβ demonstrates a diurnal pattern. We also determined normal α- and β-processing of APP in the human CNS and assessed whether AD pathology is associated with alterations in APP processing.

The regulation of APP by α- and β-secretase over time, including potential dynamic changes of sAPPα and sAPPβ within an individual, has not been previously evaluated, although Aβ diurnal activity has been described in healthy, young human participants [7]. We recently demonstrated that both in CSF [9] and in plasma [10], the physiological Aβ diurnal fluctuation described in young participants diminishes significantly with increasing age, but is not further decreased in amyloidosis. Further, previous studies in mice indicated that sleep regulation may play a critical role in the risk and development of AD [8], but more recent findings indicate that it may be Aβ aggregation that disrupts both the sleep-wake cycle and Aβ diurnal fluctuation [21]. For example, longitudinal studies have found a strong relationship between sleep circadian patterns, as well as sleep disordered breathing and risk of mild cognitive impairment and AD [22]–[23]. Therefore, we sought to determine the relationship between α- and β- processing pathways in individuals over time, and also determine if APP regulation contributes to Aβ circadian patterns.

In the YNC group, we found that sAPPα, sAPPβ, Aβ_40_, and Aβ_42_ concentrations were dynamic over 36 hours, with diurnal patterns. The lowest concentrations were in the morning (approximately 9:00 A.M.), and the concentrations peaked in the evening, approximately12 hours later. This suggests that in the YNC group, dynamic changes in these protein levels were due to dynamic changes in APP availability, whether by its production (transcription or translation) or transport to the site of processing (i.e. axonal transport). Amyloid-β also demonstrated a diurnal pattern with a peak and trough approximately three hours after sAPPα and sAPPβ. This suggests that APP diurnal availability likely plays a role in Aβ diurnal patterns.

Diurnal patterns of sAPPα and sAPPβ were diminished in the Amyloid**−** group. Aβ_42_ did not show any significant diurnal pattern in the Amyloid− group similarly to prior work from our laboratory [9]. However, whereas our present work did not show a diurnal pattern of Aβ_40_ in the Amyloid− group, there was a slight, but significant diurnal pattern observed in [9]. Potential reasons for this discrepancy include different ELISA assays employed for the different studies. Both Aβ ELISA assays from [9] used 3D6 as detection antibodies, and capture antibodies were 2G3 (anti-Aβ_40_) and 21F12 (anti-Aβ_42_). These are fairly common Aβ antibodies, and those assays provided lower intra-sample CV of duplicates than the antibodies we used for Aβ in this study. More noisy data may have contributed to slightly differing results. Further, although several of our participants in the two studies overlapped, many participants were not from the same dataset as [9]. Lastly, [9] had more variable sample size among groups (YNC = 20, Amyloid− = 15. Amyloid+ = 11), whereas our groups were more balanced (YNC = 13; Amyloid− = 15; Amyloid+ = 14). Taken together, these findings indicate that with age there is a loss of APP dynamics or availability, which results in the noted loss of not only sAPP, but also Aβ diurnal patterns. It was recently reported that sleep facilitates Aβ clearance [24], thus the physiological tightly-regulated diurnal patterns of Aβ may diminish with age due to an increase in sleep fragmentation that is common in normal aging [25] or by a general increased Aβ production due to wakefulness [8].

Lack of a diurnal pattern of sAPPα and sAPPβ was exhibited to a similar extent in the Amyloid+ group as was seen in the Amyloid**−** group. However, the diurnal patterns in Aβ_40_ and Aβ_42_ were even more significantly diminished in the Amyloid+ group than was seen in the Amyloid**−** group. The further marked decrease in Aβ_40_ and Aβ_42_ diurnal patterns in the presence of amyloidosis does not correspond to any decrease in sAPP diurnal patterns. This disconnect may be an effect of downstream APP cleavage events and not due to APP dynamics or availability, which seems to be the case in general aging. Potentially the extent of γ-secretase cleavage of APP, which is controlled by availability of the γ-secretase components or the γ-secretase level of activity, may play a role in diminishing the diurnal patterns of the two Aβ species we measured. Also, the build-up of Aβ plaques in the brains of those with amyloidosis may serve as a buffering system that decreases the dynamic nature of Aβ that is observed in healthy, younger humans. Although, the Amyloid+ group has a lower Aβ_42_ amplitude than YNC or Amyloid−, this result is not intended to suggest that Aβ_42_ amplitude should be added as an Alzheimer's diagnostic test. Currently, other tests (a combination of CSF Aβ_42_/tau, PIB PET, and FDG PET scanning) have good predictive outcomes for determining AD diagnosis. The potential minor additive diagnostic benefit of Aβ_42_ amplitude is questionable and would require a patient to be catheterized for 24 hours.

Further, sAPPα and sAPPβ were positively correlated in all groups. Positive correlation of the α- and β-secretase products suggests a non-competitive model of APP pathways: that the total APP availability drove changes in sAPPα and sAPPβ. Soluble APPα and sAPPβ were positively correlated with both Aβ species in YNC and elderly controls. However, the correlation between the sAPP species and Aβ_42_ was lost with amyloidosis. Prior evidence in the human CNS shows a positive sAPPα to sAPPβ correlation in individuals also suggesting non-competitive α- and β-pathways [26]–[29] However, *in vitro* studies of secretase inhibitors or activators, or genetically decreasing BACE1 (a β-secretase protein) or ADAM10 (an α-secretase protein) [30]–[36] support the hypothesis that α- and β-secretase pathways compete for the same APP pool due to inverse correlations during secretase inhibition (i.e. when processing through one pathway decreases, the processing of the alternative pathway increases). These studies suggest that there may be an inverse relationship between the α- and β- pathways in inhibitor studies, while our study shows that during physiologic APP processing in the human CNS, α- and β- processing are positively correlated.

We found that the molar ratio of sAPPα to sAPPβ was approximately 3∶1 with a shift to 2∶1 from α- to β-processing in the setting of amyloid deposition. The differences in ratios among these groups were not age-related since there was no significant difference between YNC and Amyloid− groups. Prior reports estimated α to β ratios of 10∶1 [31], [33], however, these *in vitro* estimates likely had lower β-secretase activity than is present in the CNS, since β-secretase is mostly found in the brain [12]–[14]. We further showed that on average sAPPβ/sAPPα was significantly higher in Amyloid+ participants than in Amyloid− participants and YNC; therefore, the ratio may be a useful indicator of Aβ plaque deposition. This result further supports the hypothesis that sporadic AD may be the result of an upregulation of β-secretase processing of APP, with respect to α-secretase. Our results are consistent with recent findings of increased CSF sAPPβ in the presence of decreased Aβ_42_ and increased tau [26]–[27]. However, some reports indicate increased sAPPα [28] while others show no difference [26]–[27], similarly to our findings. Recently, it was reported that neither sAPPα, nor sAPPβ, measured from CSF by both ELISA and mass spectrometry, was altered in AD [32]. This parallels results of an ELISA study from a decade earlier that also showed no difference in sAPPα, nor in sAPPβ, when healthy controls were compared to sporadic AD patients [37]. None of these groups, however, reported sAPP metabolite ratios. To summarize, amyloidosis, and not age, was associated with a constitutive change in α- to β- processing of APP among individuals.

In conclusion, in our study we report diurnal dynamics of APP metabolites diminished with age, and, only for Aβ, were further attenuated with amyloidosis. These results may explain some possible confounding factors of other studies that have measured sAPPα, sAPPβ, Aβ_40_, and Aβ_42_ levels in CSF collected at a single time point from AD versus non-AD participants. This may clarify the discrepancy in results and the wide range of concentrations of APP metabolites presented by various groups. We also indicate that taking a ratio of sAPPβ/sAPPα may correct for these inconsistencies. Further, we demonstrated that there is a positive correlation among soluble APP metabolites, which diminishes with amyloidosis. This dissociation is probably due to CSF Aβ_42_ levels in AD no longer being representative of APP processing due to the sequestering of Aβ, particularly Aβ_42_, in plaques.

Advantages of this study included that the samples were obtained from the human CNS in three different participant groups and total protein concentrations showed stability over time in the older groups. Fewer than half of the YNC had total protein data available, and this, along with high inter-subject variability, does not allow us to state conclusively whether a diurnal pattern of total protein does or does not exist in the whole YNC group. However, the similar diurnal patterns among APP metabolites seem to indicate that CSF APP dynamics are likely independent of CSF total protein levels. Nevertheless, we did not directly measure α- and β-secretase activities or production rates of APP metabolites. Thus, our study does not answer the question of what causes APP to rise and fall in a diurnal pattern, although possibilities include transcription, translation, or transport. Future studies into APP processing pathways, including production rates of APP and α- and β-secretases may be useful to inform about causes of APP dynamics.

## Supporting Information

Figure S1 **Specificity and selectivity of the sAPPα ELISA.** Titration curves of sAPPα and sAPPβ standards were run on the sAPPα ELISA assay. The OD values from the CSF samples fell well above baseline, and within the linear range of the sAPPα standard curve. This demonstrates that this assay is sensitive enough to measure sAPPα from the biological samples in this study. The sAPPβ standard curve's OD values were zero, even at the highest concentration of 300 ng/mL, which indicates that sAPPβ does not cross-react with the sAPPα assay.
(TIFF)

Figure S2 **Specificity and selectivity of the sAPPβ ELISA.** Titration curves of sAPPα and sAPPβ standards were run on the sAPPβ ELISA assay. The OD values from the CSF samples fell well above baseline, and within the linear range of the sAPPβ standard curve. This demonstrates that this assay is sensitive enough to measure sAPPβ from the biological samples in this study. The optical density (OD) for the sAPPβ standard of 8.5 ng/mL was approximately the same as the OD value for the sAPPα standard at a concentration of 300 ng/mL. This indicates that this ELISA is approximately 35-fold more selective for sAPPβ than for sAPPα. Thus, any cross-reactivity is negligible.
(TIFF)

Figure S3 **No diurnal pattern in total CSF protein concentrations of Amyloid− and Amyloid+ groups.** Participants' total protein concentrations in CSF over 36 hours were determined by using a micro BCA assay. For each participant group, the mean total protein concentration for each hour was calculated and plotted. Cosinor fits were applied to each group's hourly mean total protein concentration. A significant cosinor fit was found in the YNC group (n = 6), with an amplitude 4.5% (95% CI: −6.1% to −2.9%). No significant diurnal patterns were apparent in the Amyloid− group (n = 6; 95% CI: −1.4% to +8.6%) and the Amyloid+ group (n = 5; 95% CI: −8.4% to +1.4%).
(TIFF)

Figure S4 **sAPPβ/sAPPα ratios determined from 36 hour time-course.** We measured the sAPPβ/sAPPα ratio for each individual based on that participant's sAPPβ and sAPPα concentrations over the 36 hour time-course. Individual ratios were calculated and averaged within participant groups. Student's *t*-test was used and graphs show 95% Confidence Interval error bars. **A**) Mean sAPPβ/sAPPα ratio was calculated for each participant using that participant's 36 hour mean sAPPβ concentration and 36 hour mean sAPPα concentration. Individual ratios were averaged in their respective participant groups. The group-averaged mean sAPPβ/sAPPα ratio is significantly higher in YNC than in Amyloid− (*p = 0.03) or in Amyloid+ (*p = 0.03). No significant difference was detected between the group-averaged mean sAPPβ/sAPPα ratio of the Amyloid− and Amyloid+ groups (*p* = 0.92). **B**) Mesor sAPPβ/sAPPα ratio was calculated for each participant using the sAPPβ mesor value (determined from the cosinor fit of that participant's 36 hour sAPPβ concentrations) and the sAPPα mesor value (determined from the cosinor fit of the 36 hour sAPPα concentrations). The mesor sAPPβ/sAPPα ratio is significantly higher in YNC than in Amyloid− (*p = 0.03) or in Amyloid+ (*p = 0.03). No significant difference was detected between the mesor sAPPβ/sAPPα ratio of the Amyloid− and Amyloid+ groups (*p* = 0.93).
(TIFF)
