# Supplementary figures and images for: Diurnal Patterns of Soluble Amyloid Precursor Protein Metabolites in the Human Central Nervous System
Source: PLoS One. 2014 Mar 19;9(3):e89998. doi: 10.1371/journal.pone.0089998 (PMC3960093; doi:10.1371/journal.pone.0089998)

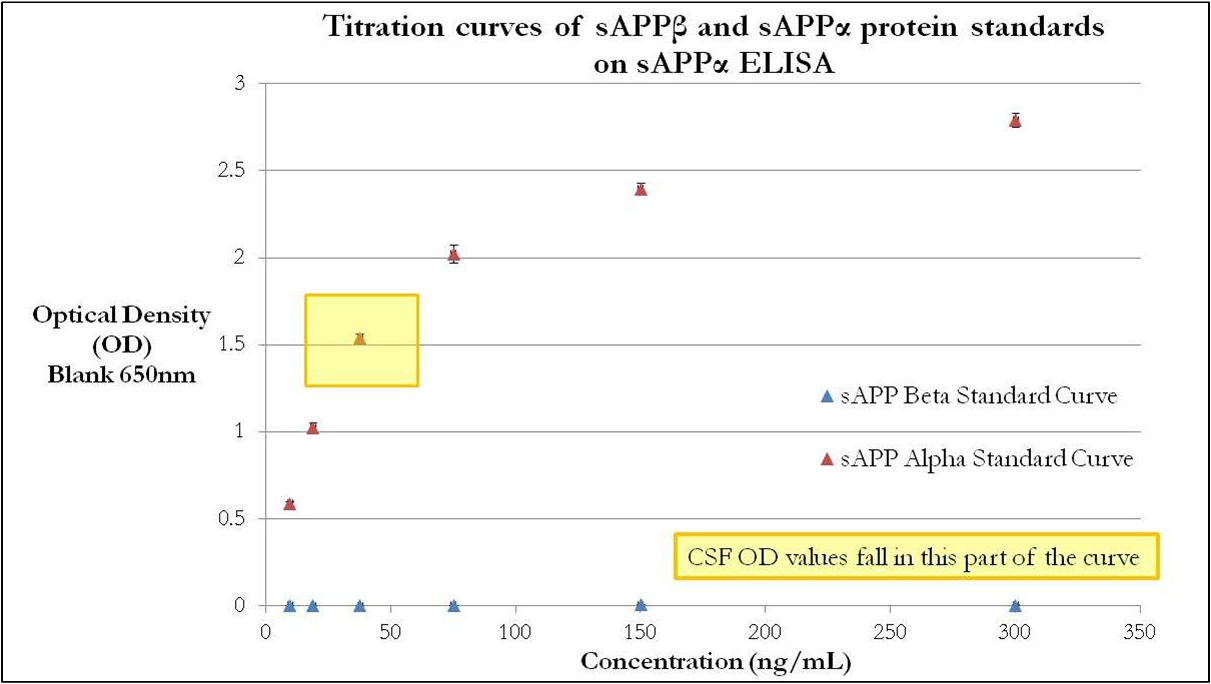

Supplement: Figure S1 — Specificity and selectivity of the sAPPα ELISA. Titration curves of sAPPα and sAPPβ standards were run on the sAPPα ELISA assay. The OD values from the CSF samples fell well above baseline, and within the linear range of the sAPPα standard curve. This demonstrates that this assay is sensitive enough to measure sAPPα from the biological samples in this study. The sAPPβ standard curve's OD values were zero, even at the highest concentration of 300 ng/mL, which indicates that sAPPβ does not cross-react with the sAPPα assay. (TIFF) [file pone.0089998.s001.tiff]

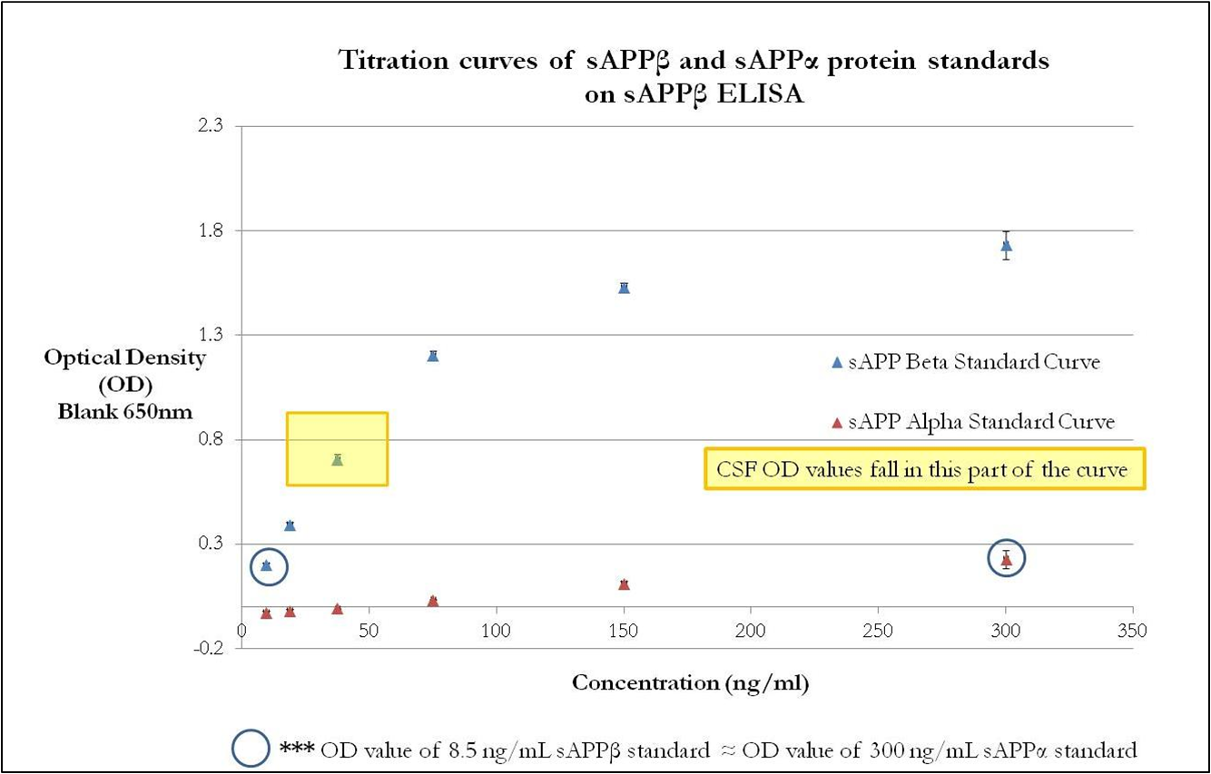

Supplement: Figure S2 — Specificity and selectivity of the sAPPβ ELISA. Titration curves of sAPPα and sAPPβ standards were run on the sAPPβ ELISA assay. The OD values from the CSF samples fell well above baseline, and within the linear range of the sAPPβ standard curve. This demonstrates that this assay is sensitive enough to measure sAPPβ from the biological samples in this study. The optical density (OD) for the sAPPβ standard of 8.5 ng/mL was approximately the same as the OD value for the sAPPα standard at a concentration of 300 ng/mL. This indicates that this ELISA is approximately 35-fold more selective for sAPPβ than for sAPPα. Thus, any cross-reactivity is negligible. (TIFF) [file pone.0089998.s002.tiff]

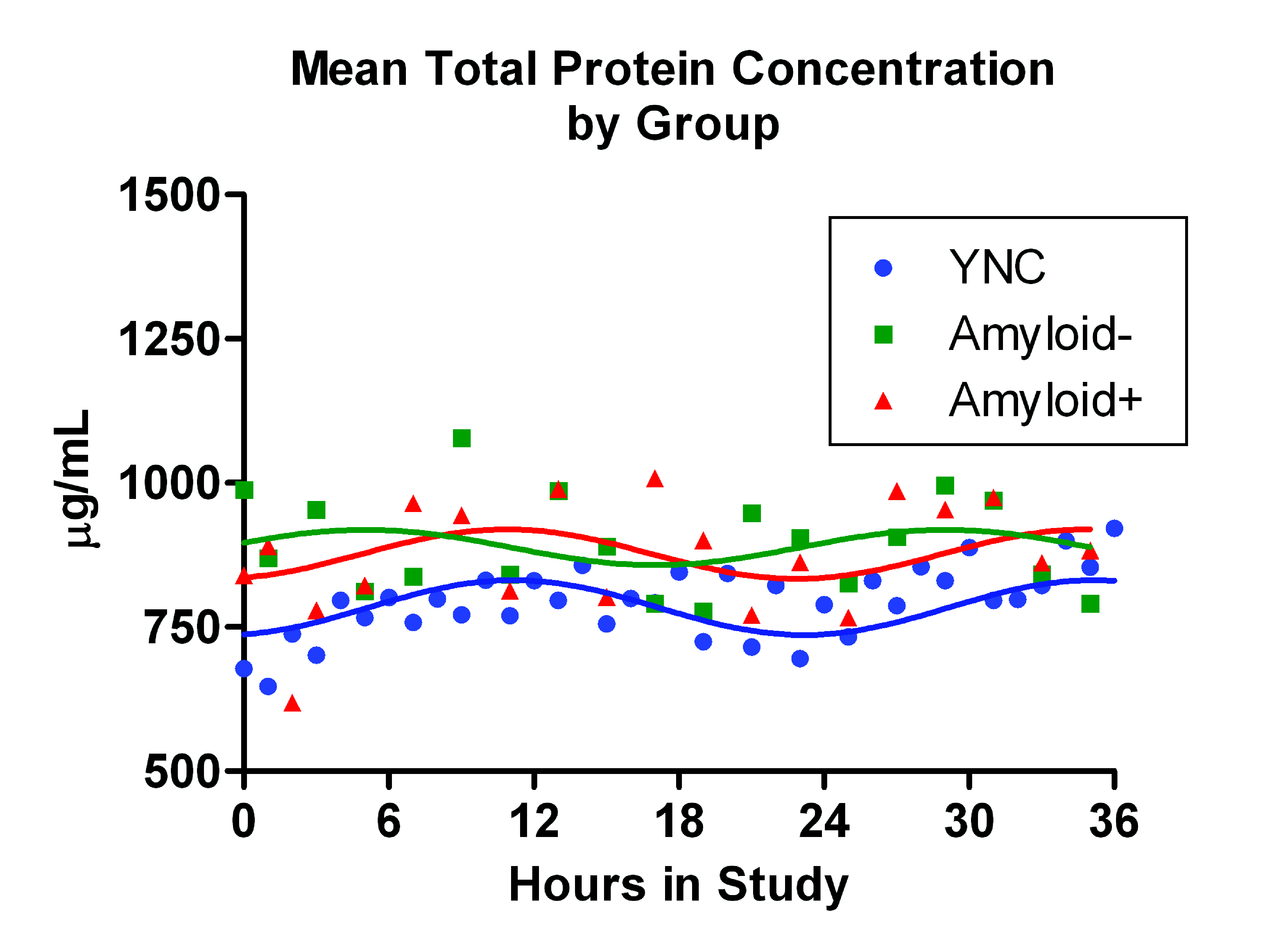

Supplement: Figure S3 — No diurnal pattern in total CSF protein concentrations of Amyloid− and Amyloid+ groups. Participants' total protein concentrations in CSF over 36 hours were determined by using a micro BCA assay. For each participant group, the mean total protein concentration for each hour was calculated and plotted. Cosinor fits were applied to each group's hourly mean total protein concentration. A significant cosinor fit was found in the YNC group (n = 6), with an amplitude 4.5% (95% CI: −6.1% to −2.9%). No significant diurnal patterns were apparent in the Amyloid− group (n = 6; 95% CI: −1.4% to +8.6%) and the Amyloid+ group (n = 5; 95% CI: −8.4% to +1.4%). (TIFF) [file pone.0089998.s003.tiff]

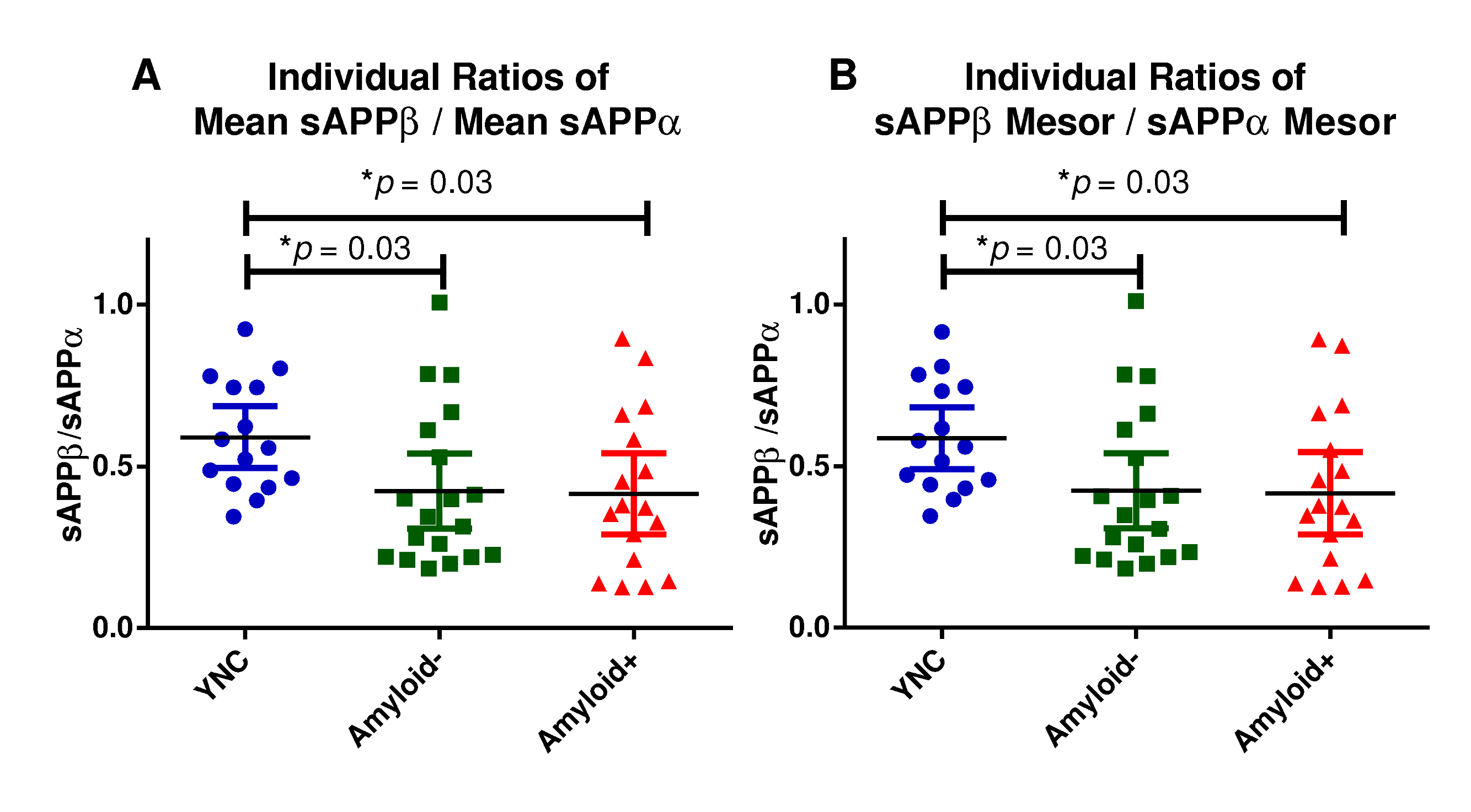

Supplement: Figure S4 — sAPPβ/sAPPα ratios determined from 36 hour time-course. We measured the sAPPβ/sAPPα ratio for each individual based on that participant's sAPPβ and sAPPα concentrations over the 36 hour time-course. Individual ratios were calculated and averaged within participant groups. Student's t-test was used and graphs show 95% Confidence Interval error bars. A) Mean sAPPβ/sAPPα ratio was calculated for each participant using that participant's 36 hour mean sAPPβ concentration and 36 hour mean sAPPα concentration. Individual ratios were averaged in their respective participant groups. The group-averaged mean sAPPβ/sAPPα ratio is significantly higher in YNC than in Amyloid− (*p = 0.03) or in Amyloid+ (*p = 0.03). No significant difference was detected between the group-averaged mean sAPPβ/sAPPα ratio of the Amyloid− and Amyloid+ groups (p = 0.92). B) Mesor sAPPβ/sAPPα ratio was calculated for each participant using the sAPPβ mesor value (determined from the cosinor fit of that participant's 36 hour sAPPβ concentrations) and the sAPPα mesor value (determined from the cosinor fit of the 36 hour sAPPα concentrations). The mesor sAPPβ/sAPPα ratio is significantly higher in YNC than in Amyloid− (*p = 0.03) or in Amyloid+ (*p = 0.03). No significant difference was detected between the mesor sAPPβ/sAPPα ratio of the Amyloid− and Amyloid+ groups (p = 0.93). (TIFF) [file pone.0089998.s004.tiff]
